# Surgical outcome of laparoscopic colectomy for colorectal cancer in obese patients: A comparative study with open colectomy

**DOI:** 10.3892/ol.2013.1508

**Published:** 2013-08-02

**Authors:** YANTAO CAI, YIMING ZHOU, ZHENYANG LI, JIANBIN XIANG, ZONGYOU CHEN

**Affiliations:** Department of General Surgery, Huashan Hospital Affiliated to Fudan University, Shanghai 200040, P.R. China

**Keywords:** colorectal, obesity, laparoscopic, open

## Abstract

The aim of the present study was to assess the short-term outcome and survival time of 166 obese patients who received laparoscopic and open colectomy for colorectal cancer (CRC) between January 2007 and December 2012. All 166 patients included in the study had a BMI >28. Laparoscopic or open colectomy procedures were performed on 64 and 102 patients, respectively. The short-term outcome and post-operative survival rates were compared. The patient characteristics were similar between the two groups. Laparoscopic colectomy correlated with an increased duration of surgery compared with open colectomy (183 vs. 167 min, respectively; P<0.05) but intraoperative blood loss was decreased (168 vs. 188 ml, respectively; P<0.05). Hospitalization costs were slightly higher following the laparoscopic procedure compared with open surgery, but this was affordable for the majority of patients (¥56,484 vs. ¥56,161, respectively; P<0.05). The incidence of wound infection (17 vs. 31%; P<0.05) and abdominal abscess rates (6 vs. 18%; P<0.05) were reduced in the laparoscopic group compared with the open group. Pathological characteristics were identified to be similar and no significant differences were identified in overall (log-rank test; P=0.85) and disease-free (log-rank test; P=0.85) survival between the two types of surgery (log-rank test; P=0.76). The current retrospective study demonstrated an improved short-term outcome in laparoscopic colectomy for CRC patients with a BMI >28 compared with patients who underwent the open procedure. Laparoscopic colectomy is technically and oncologically safe and must be popularized in obese CRC patients.

## Introduction

Obesity is an increasing social problem with a significant effect on an individuals health that has subsequently been defined as a disease by the World Health Organization (WHO) (1). Due to changing diets and growing socioeconomic prosperity, the prevalence of overweight and obese individuals in China has increased to 29.9% according to data from the China National Nutrition and Health Survey in 2002 (2). It has been proven that obesity is an independent risk factor associated with an increased comorbidity, post-operative morbidity, risk of anesthesia and difficulties in surgery. The introduction of laparoscopic techniques initiated a new era for colorectal cancer (CRC) surgery. Previous studies have shown that a laparoscopy is less invasive with improved short-term outcomes compared with traditional open surgery (3). The anatomical development of complete mesocolic and total mesorectal excision in colon and rectal cancer, respectively, has improved oncological safety. Previously, obesity prevented the use of laparoscopic colorectal surgery, however, following improvements to the instruments and techniques involved, this procedure may now be performed in obese individuals, although it requires surgical expertise to perform the procedure safely. However, a number of studies have shown inevitably higher rates of conversion and post-operative complications in the laparoscopic surgery of obese patients compared with non-obese patients (4–6).

The present study was conducted to compare the short-term outcome and survival time of obese patients receiving laparoscopic or open colectomy for CRC. The results are likely to aid in the selection of a suitable surgical approach for obese CRC patients.

## Materials and methods

### Eligibility

Between January 2007 and December 2012, 166 patients with a pre-operative BMI >28 underwent laparoscopic or open colectomy for CRC in the Department of General surgery, Huashan Hospital (Shanghai, China). The exclusion criteria were as follows: i) Patients who had a tumor that could not be resected radically; ii) patients who had undergone abdominoperineal resection; iii) a history of previous gastrointestinal surgical history; iv) patients who had received neoadjuvant chemoradiotherapy prior to surgery; v) patients who had undergone emergency surgery; and vi) cases with incomplete medical records. This study was approved by the ethics committee of Huashan Hospital Affiliated to Fudan University (Shanghai, China). Written informed consent was obtained from the patient’s family.

### Data collection methods

Data were obtained from the patient medical record database of Huashan Hospital (Shanghai, China), including: i) Patient characteristics, i.e., gender, age, BMI, tumor location, American Society of Anesthesiologist classification and pre-operative complications; ii) intraoperative data, i.e., operating time, blood loss and conversion to open procedure; iii) post-operative data, i.e., first bowel movement, number of days to initiation of fluid diet, drainage length and the length and cost of hospitalization; and iv) post-operative complications, i.e., wound infection, anastomotic leak, pulmonary complications, abdominal abscess, ileus, chyle leakage, hemorrhage, deep vein thrombosis and repeat surgery. Length of hospitalization was defined as the period between admittance and discharge. Patients were followed at outpatient clinics every 3 months for the first 2 years and every 6 months thereafter, in addition to phone calls, mail and e-mail follow-ups.

### Surgical procedure

Patients with pre-operative complications were administered with appropriate therapy prior to surgery under consultation. For the laparoscopic surgery, four or five trocars were inserted whilst pneumoperitonium pressure was maintained at 10–14 mmHg. The exact surgical type was determined according to the location of the tumor and via intraoperative detection. The principle of obtaining a radical cure was followed during surgery. Staplers were applied to achieve anastomosis in the two groups and drainage was used routinely at the correct locations. Patients with T3/T4 or lymph node metastasis received post-operative systemic adjuvant chemotherapy according to the National Comprehensive Cancer Network (NCCN) Clinical Practice Guidelines in Rectal Cancer, 2011.

### Statistical analysis

The statistical analysis was performed using the SPSS 19.0 statistical software package (SPSS Inc., Chicago, IL, USA). Continuous data are presented as the mean± SD or as indicated. Quantitative variables were analyzed by Student’s t-test, and χ^2^ and Fisher’s exact tests were used to analyze group comparisons where appropriate. Overall and disease-free survival rates were analyzed using Kaplan-Meier curves and evaluated with the log-rank test. P<0.05 was considered to indicate a statistically significant difference.

## Results

A total of 166 eligible patients who underwent laparoscopic or open colectomy for CRC between 2007 and 2012 were distributed into two groups according to the type of surgery performed. A total of 64 patients formed the laparoscopic group and 102 patients formed the open group. No significant differences in demographic data were identified between the two groups (Table I).

Table II presents the intra- and post-operative data. The duration of surgery was higher in the laparoscopic group compared with the open group (183 vs. 167 min, respectively; P<0.05), in addition to a significantly reduced intraoperative blood loss (168 vs. 188 ml, respectively; P<0.05). Patients who underwent laparoscopic colectomy had marginally higher overall hospitalization costs compared with the patients from the open group (¥56,484 vs. ¥56,161, respectively; P<0.05). The post-operative rates of wound infection and abdominal abscess were lower in the laparoscopic group compared with the open group (17 vs. 31%; and 6 vs. 18%, respectively; both P<0.05) and no significant differences in additional post-operative complications were identified between the two groups.

As demonstrated in Table III, conversion to open surgery was required in 3 cases (4.7%) from the laparoscopic group due to severe intra-abdominal adhesion, obesity-hindering vision and bleeding. In addition, there was one repeat surgery case in the laparoscopic group due to a small bowel obstruction and two cases in the open group due to anastomotic hemorrhage and leakage (Table IV). An additional patient with comorbidity of cardiac insufficiency developed acute heart failure and succumbed to cardiac complications on day 28 following open radical resection of sigmoid colon cancer. No significant differences in pathological characteristics were identified between the two groups, as shown in Table V.

**Figure 1:**
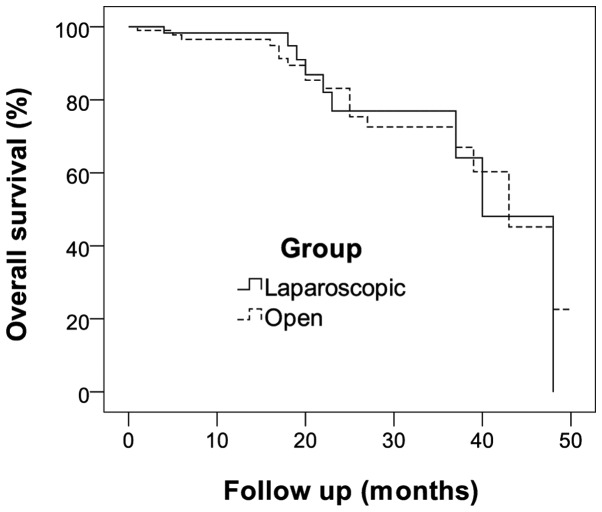
Overall survival rate of laparoscopic and open colectomy in colorectal cancer (CRC) patients with a BMI >28. Kaplan-Meier analysis (log-rank test, P=0.85).

**Figure 2:**
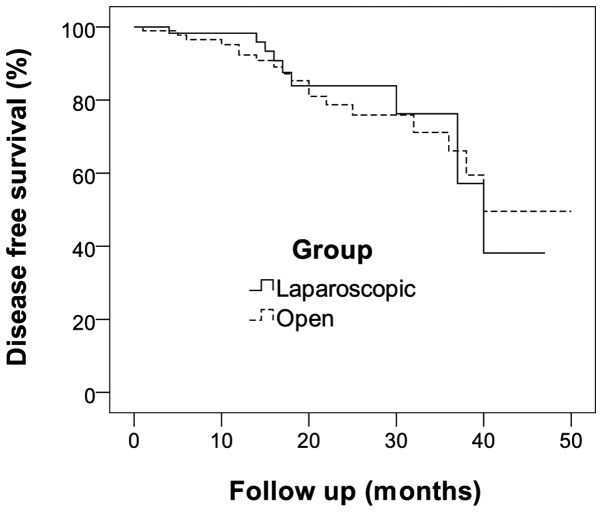
Disease-free survival rate of laparoscopic and open colectomy in colorectal cancer (CRC) patients with a BMI >28. Kaplan-Meier analysis (log-rank test, P=0.76).

Patients were followed until mortality or for 1–50 months. The median follow-up period was 17 months by means of outpatient services, phone calls and mail/e-mail. At the end-point of follow-up, the survival rate was 133/166 patients (80.1%). Local recurrence was identified in 14 patients (8.8%) and 34 patients developed distal metastases during follow-up, including 20 hepatic, 8 pulmonary, 4 hepatopulmonary, 1 osseous and 1 brain metastasis. During the follow-up period, tumor-related mortalities occurred in 33 patients (19.9%). The overall survival time in the laparoscopic and open groups was 38.7±2.7 and 38.7±2.0 months, respectively (Fig. 1) and the disease-free survival time was 37.3±2.5 and 38.7±2.0 months, respectively (Fig. 2). Results of the log-rank test demonstrated that the overall and disease-free survival rates for the laparoscopic and open groups were equivalent.

## Discussion

A number of previous studies have shown that laparoscopic colectomy is technically and oncologically safe (7,8) with reduced invasive manipulation and improved intra-abdominal vision and post-operative recovery compared with traditional open surgery. Due to limitations in the technique, instruments and surgical experience, obese individuals were not previously treated using laparoscopic colectomy. The increased levels of fat tissue in obese patients affects surgery by hindering visualization, dissection of the tissue planes and ligation of the vessels (9). Due to increasing surgical experience, a previous study reported the optimistic outcome of practicing laparoscopic colectomy on obese patients, although the surgery continues to be technically difficult (6). Therefore, with surgical safety and oncological clearance guaranteed, the application of a laparoscopic technique for colectomy in obese patients is promising.

BMI converts obesity into a numerical concept and is commonly used to define the level of obesity in patients. In accordance with the classification of the WHO, obesity is defined as a BMI >30 kg/m^2^ and this figure is widely accepted in Western countries (1). However, in China, the criteria for obesity is lower with a BMI >28 kg/m^2^(2) due to the analysis of data collected via a large census of the Chinese population in the 1990s. In addition, other results have revealed that Asian Pacific populations, including that of China, have an elevated risk for obesity-related diseases at a lower BMI when compared with that of Caucasians (10). Therefore, the Chinese criteria for obesity, BMI>28, was adopted for the present study (11).

Previous studies have identified that obesity functions as an independent factor leading to a poorer short-term surgical outcome and long-term prognosis (12). In laparoscopic and open surgery, obesity correlates with an increased pre-operative comorbidity, volume of blood loss, surgical difficulty and post-operative complications. In addition, obesity shows a significantly higher rate of conversion to open surgery when compared with normal BMI patients (4). Obese individuals represent a unique subset in the treatment of CRC since obese patients are likely to suffer from increased operative difficulties, conversion rates and post-operative morbidities. These negative factors are capable of counteracting the advantages of undergoing laparoscopic surgery. Therefore, the decision between laparoscopic colectomy and open surgery for obese patients in accordance to an analogy of short-term surgical outcome in the normal population is not reliable. Few clinical studies have analyzed the short-term surgical outcomes between laparoscopic and open colectomy in obese patients, therefore, the aim of the current study was to investigate the surgical outcome of laparoscopic colectomy for CRC in obese patients. The results are likely to improve the selection of a suitable surgical approach for this subset of patients.

A previous study compared the short-term surgical outcomes of laparoscopic and open colorectal surgery in the population as a whole, including obese patients, and indicated that the laparoscopic technique is associated with a prolonged surgical duration but decreased intraoperative blood loss (13). These results are similar to those of the current study, which focused on obese patients, as when compared with the open group, the laparoscopic group also showed a significantly higher mean duration of surgery (183 vs. 167 min; P<0.05) and decreased blood loss (168 vs. 188 ml, respectively; P=0.02). Similarly, a previous study by Balentine *et al* analyzed the records of 155 obese patients (BMI >30) and showed decreased intraoperative blood loss and a higher surgical duration in the laparoscopic group compared with the open group (14). Previous studies have identified that the increased difficulty of mesentery dissection and main vessel ligation in obese patients contributes to a prolonged surgical duration (15). These difficulties in manipulation are increased in obese patients due to restricted intra-abdominal space for surgery and a fatty mesentery (15). Decreased blood loss was observed in the laparoscopic group of the current study when compared with that of the open group (168 vs. 188 ml, respectively; P=0.02); this is likely to be due to the ability to magnify the field of view under the laparoscope. Adjusting the angle of the 30º laparoscope may improve the surgeon’s vision in a comfortable position allowing dissection and ligation with increased precision. This reduces the risk of main vessel injury. Mobilization of the intestines was avoided due to the satisfactory conditions of the anatomy of the surgical region. However, despite a significant difference in the mean blood loss (20 ml), the decreased volume recorded in the laparoscopic group resulted in no differences in the requirements for transfusion. Another study has shown that a decreased requirement for transfusion may correlate with increased post-operative morbidity (16).

In addition, no significant differences between the number of days to the first bowel movement, initiation of fluid diet, indwelling drainage and hospitalization were identified between the laparoscopic and open groups, despite slight decreases observed in the laparoscopic group. However, the rate of wound infection was significantly decreased in the laparoscopic group compared with the open group (17 vs. 31%, respectively; P=0.047). The increased thickness of the adipose tissue layer in obese patients usually leads to a higher incidence rate of fat liquefaction, incision infection and impaired wound healing (17). Methods, including drainage and changing the dressing of the wound, are required to control infection and therefore the length and cost of hospitalization increases. Previous studies have identified that the length of the incision correlates positively with the risk of wound infection. The retractor used in open surgery exposes the surgical field by separating the incision edges. However, this is capable of inducing hypoxia at the incision site, which increases the risk of wound infection (18). In laparoscopic surgery, a pneumoperitoneum is used to expose the surgical field instead of a retractor. A pneumoperitoneum spreads the pressure on the abdominal wall and subsequently reduces the risk of hypoxia in the tissue compared with open surgery. A shorter incision is made to remove the resected tumor or perform anastomosis in specific types of laparoscopic colectomy, and in addition, the incision may be eliminated completely in specific anterior resections via a pull-through technique (19). The significantly decreased rate of wound infection following laparoscopic colectomy may be due to decreased pressure on the incision and a shorter incision length.

Abdominal abscesses are a serious complication following surgical practice and the complex nature of a number of CRC cases of these lesions may lead to their formation. Diagnosis and treatment is often delayed due to atypical clinical signs (20). The decreased incidence of abdominal abscesses in the laparoscopic group compared with the open group (6 vs. 18%, respectively; P=0.037) observed in the current study has not been previously reported (14). Anastomotic leakage has previously been confirmed as the most common cause of abdominal abscesses, however, the results of the present study showed no significant differences between the incidence of abscess and this cause (21). Therefore, it is hypothesized that the decreased incidence of abdominal abscess results from reduced mobilization and dissection of the mesentery during laparoscopic surgery compared with open surgery, and this is likely to aid the prevention of injury to fat tissue and abscess formation.

Hospitalization costs in the present study were defined as the total expenses between admission and discharge, including the costs of surgery, examination and the treatment of complications. The average hospitalization cost in the laparoscopic group was ¥56,484, which was an additional ¥323 compared with the cost in the open group (P<0.05). In the early development of laparoscopic colectomy, overall costs were reported to be higher than that of traditional open surgery (22). A prospective cost analysis in the UK compared the costs of laparoscopic and open colorectal surgery and revealed that surgery costs were higher in laparoscopic surgery when compared with that of open surgery (£2,049 vs. £1,263, respectively; P<0.001), however, significantly lower hospitalization costs following laparoscopic surgery (£1,807, vs. £3,468, respectively; P<0.001) were also identified. Therefore, the overall costs were almost equivalent (£3,875 vs. £4,383; P=0.308) (23). In addition, a previous study performed by the Cleveland Center indicated that no significant differences were identified between the overall costs for laparoscopic and open surgery ($4,003 vs. $4,037, respectively; P=0.14) (24). However, in the current study, a significant difference was identified between the overall hospitalization costs in laparoscopic and open surgery, however, the ¥323 difference was affordable for the majority of patients. The use of disposable instruments contributes to the increased surgical costs of laparoscopic colectomy compared with open surgery. However, the advantages of laparoscopic surgery in the post-operative period include reduced complications and a faster recovery. Therefore, the costs of hospitalization and mortality treatment may be decreased. The current study identified higher rates of pre- and post-operative complications in obese patients, increasing overall hospitalization cost. The rates of post-operative wound infection and abdominal abscess formation were decreased in the laparoscopic group. This was hypothesized to reduce the requirement for antibiotics and additional supportive treatment and therefore improve the cost gap between laparoscopic and open colectomy. A similar overall cost and an improved short-term outcome indicates a promising future for laparoscopic colectomy in obese CRC patients.

The post-operative pathological characteristics were similar in the two groups of the present study and no significant differences were identified between the number of harvested lymph nodes, consistent with previous studies (8,25). In addition, no significant differences between overall and disease-free survival were identified during the follow-up period between the two groups. These results indicate that laparoscopic surgery does not affect survival rate and recurrence, consistent with results of a study performed by Nelson *et al*(26). A number of studies have reported higher survival rates at specific stages of CRC following a laparoscopic procedure compared with open surgery (13,27). At present, it is widely accepted that the long-term oncological outcome for laparoscopic CRC resection is not inferior to the traditional open surgery approach, and the results of the present study identified this in patients with a BMI >28.

To conclude, the results of the present study indicate that laparoscopic colectomy is technically and oncologically safe for treating obese CRC patients, and may represent a promising choice of surgery in clinical practice.

## Figures and Tables

**Table I:** Summary of patient characteristics and comparison between the open and laparoscopic groups.

	Open	Laparoscopic	P-value
Patients, n	102	64	
Gender, n			0.502
Male	60	41	
Female	42	23	
Age, years	63.1±11.5	64.4±13.1	0.258
BMI	29.69±1.51	29.28±1.25	0.567
Tumor location, n			0.429
Cecum	9	7	
Ascending colon	28	13	
Transverse colon	12	5	
Descending colon	7	7	
Sigmoid colon	33	18	
Rectum	13	14	
ASA class, n			0.196
1	36	18	
≥2	66	46	
Pre-operative complications, n			
Cardiovascular	48	31	0.753
Endocrine	19	12	0.312

Data are mean ± standard deviation unless otherwise stated. ASA, American Society of Anethesiologists.

**Table II:** Intraoperative data and post-operative complications.

	Open	Laparoscopic	P-value
Patients, n	102	64	
Operating time, min	167±32	183±55	<0.050
Estimated blood loss, ml	188±83	168±106	0.023
First bowel movement, days (range)	4 (2–8)	3 (1–10)	0.225
Initiation of fluid diet, days (range)	4 (1–7)	3 (1–23)	0.776
Drainage length, days (range)	7 (4–20)	6 (3–34)	0.369
Length of hospitalization, days (range)	15 (8–55)	12 (6–39)	0.201
Hospitalization costs, ¥	56161±16662^a^	56484±11514^a^	<0.050
Post-operative complications, n (%)			
Wound infection	32 (31)	11 (17)	0.047
Anastomotic leak	6 (6)	3 (5)	1.000^b^
Pulmonary complications	8 (8)	2 (3)	0.322^b^
Abnominal abscess	18 (18)	4 (6)	0.037^b^
Ileus	5 (5)	5 (8)	0.511^b^
Chyle leakage	3 (3)	2 (3)	1.000^b^
Hemorrhage	1 (1)	0 (0)	1.000^b^
Deep vein thrombosis	1 (1)	0 (0)	1.000^b^
Repeat surgery	2 (2)	1 (2)	1.000^b^

a USD 1.00 = RMB 6.23 on January 7th 2013;

b Fisher’s exact test.

Data are presented as mean ± standard deviation unless otherwise stated.

**Table III:** Cases of conversion from laparoscopy to open colectomy.

Age, years	Gender	BMI	Location of cancer	Pre-operative complications	Reason for conversion of surgery, min	Duration ml	Blood loss,
84	Male	28.95	Transverse colon	Diabetes and Atrial fibrillation	Severe intra-abdominal adhesion	180	150
68	Male	30.77	Ascending colon	Diabetes	Obesity-hindering vision	220	300
66	Female	28.40	Descending colon	Hypertension	Bleeding	300	400

**Table IV:** Repeat surgery at 30 days.

Age, years	Gender	BMI	Comorbidity	First surgery	Reason for repeat surgery
80	Male	29.57	Hypertension and Diabetes	Open anterior resection	Anastomotic hemorrhage
58	Male	30.46	None	Open right hemicolectomy	Anastomotic leakage
82	Male	28.86	Diabetes	Laparoscopic anterior resection	Small bowel obstruction

**Table V:** Pathological characteristics.

Characteristics	Open	Laparoscopic	P-value
T, n			0.213
1–2	15	15	
3–4	87	49	
N, n			0.312
−	69	48	
+	33	16	
No. of lymph nodes harvested	11.81±5.40	11.68±5.69	0.921
TNM stage, n			0.412
I	14	13	
II	55	35	
III	33	16	

T, size and/or extension of primary tumor; N, regional lymph nodes. Data are presented as mean ± standard deviation unless otherwise stated.
